# Spider silks: recombinant synthesis, assembly, spinning, and engineering of synthetic proteins

**DOI:** 10.1186/1475-2859-3-14

**Published:** 2004-11-16

**Authors:** Thomas Scheibel

**Affiliations:** 1 Department of Chemistry, Lehrstuhl für Biotechnologie, Technische Universität München, Lichtenbergstr. 4, 85747 Garching, Germany

## Abstract

Since thousands of years humans have utilized insect silks for their own benefit and comfort. The most famous example is the use of reeled silkworm silk from *Bombyx mori *to produce textiles. In contrast, despite the more promising properties of their silk, spiders have not been domesticated for large-scale or even industrial applications, since farming the spiders is not commercially viable due to their highly territorial and cannibalistic nature. Before spider silks can be copied or mimicked, not only the sequence of the underlying proteins but also their functions have to be resolved. Several attempts to recombinantly produce spider silks or spider silk mimics in various expression hosts have been reported previously. A new protein engineering approach, which combines synthetic repetitive silk sequences with authentic silk domains, reveals proteins that closely resemble silk proteins and that can be produced at high yields, which provides a basis for cost-efficient large scale production of spider silk-like proteins.

## Review

### Types of spider silk

**Figure 1:**
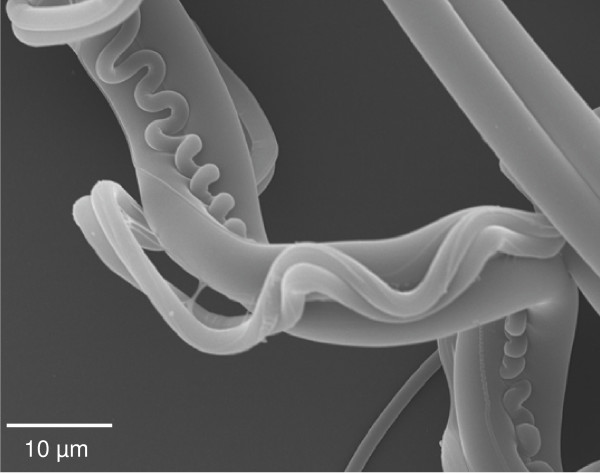
**Scanning electron microscopy of major and minor ampullate and flagelliform silks collected from the garden cross spider *Araneus diadematus. ***Silk harvested from a web was placed on Thermanox plastic cover slips (Nalgene Nunc). Samples were vacuum coated with a gold layer and analyzed with a JSM-5900LV (JEOL Ltd.) at 20 kV.

Spiders and silks always go together. Currently there are over 34,000 described species of spiders, all of which have a varied tool kit of task-specific silks with divergent mechanical properties [1-6]. Although some spiders may use silk sparingly, most make rather elaborate nests, traps and cocoons typically using more than one type of silk (Figure 1), which are produced by a wide and diverse range of glands, ducts and spigots.

Among the various spider silks the major ampullate (MA) silk, which forms the primary dragline, is extremely tough. MA silk reveals a tensile strength that is comparable to Kevlar (4 × 10^9 ^N/m^2^) coupled with a reasonable viscoelasticity (dragline 35 %, Kevlar 5 %). Spiders use dragline silk as a strong yet flexible structural element in the web, providing a framework to which other silks are attached, and as a life line when a spider is dropping off to escape an enemy. Minor ampullate (MI) silk, used for structural reinforcement in construction of the web, has a similar high tensile strength in comparison to major ampullate silk but has little elasticity [7,8]. Due to the low elasticity of MI silk it is irreversibly deforming when stretched. An orb web's capture spiral, in part composed of viscid silk formed by the flagelliform gland, which is therefore named flagelliform silk, is stretchy and can triple in length before breaking, but provides only half the tensile strength of major ampullate silk [9]. The combination of strength and stretchiness gives the capture spiral a toughness (energy to break) greater than elastin, tendon, silkworm silk, bone, synthetic rubber, Kevlar, and high-tensile steel.

### Composition and structural architecture of spider silks

Spider silks are protein polymers that display extraordinary physical properties [1-4,8], but there is only limited information on the composition of the various silks produced by different spiders. Among the different types of spider silks, draglines from the golden orb weaver *Nephila clavipes *and the garden cross spider *Araneus diadematus *are most intensely studied. Dragline silks are generally composed of two major proteins [5,10-13] and it remains unclear whether additional proteins play a significant role in silk assembly and the final silk structure. The two major protein components of draglines from *Nephila clavipes *are termed MaSp1 and MaSp2 (Major ampullate Spidroins) and from *Araneus diadematus *ADF-3 and ADF-4 (Araneus Diadematus Fibroin). The dragline silk proteins have apparent molecular masses between 180 kDa and 720 kDa depending on the conditions of analysis [14-16]. It is assumed that, based on amino acid composition, within the dragline fiber the molecular ratio between MaSp1 and MaSp2 and between ADF-4 and ADF-3 is approximately 3 to 2 [10,11,17].

**Figure 2:**
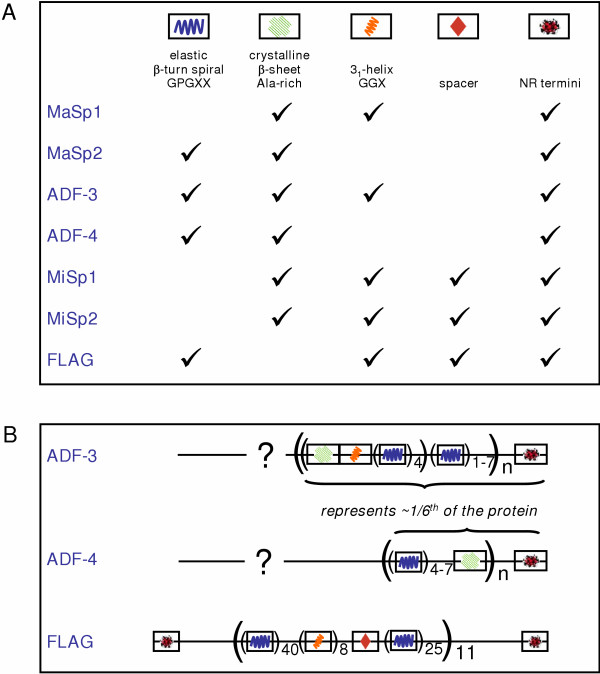
**Composition of spider silks. **(A) Structural motifs occurring within spider silks. X indicates a residue that may vary within or between proteins. The spacer represents non-repetitive but conserved regions that disrupt the glycine-rich repeats. More details on the motifs can be found in the text. (B) The sequenced cDNAs of *adf-3 *and *adf-4 *code for the shown amino acid motifs and represent approximately 1/6^th ^of the entire dragline silks. Both comprise a non-repetitive (NR) carboxyl-terminal region and a large repeat unit based on three major peptide motifs as visualized. The amino-terminal region is so far unresolved for any dragline silk. The predicted flagelliform silk protein is organized into non-repetitive (NR) amino-terminal and carboxyl-terminal regions that flank a repetitive region made up of 11 iterations of a repeating unit. Each unit contents approximately 440 amino acids. Three types of sub-repeats are present in an ensemble with the predominant unit being GPGXX.

Based on DNA analysis it could be shown that all spider silk proteins are chains of iterated peptide motifs (so called repeating units) (Figure 2). The small peptide motifs can be grouped into four major categories: GPGXX (with X often representing Q), alanine-rich stretches (A_n _or (GA)_n_), GGX, and spacers (Figure 2A). A fifth category is represented by non-repetitive (NR) regions at the amino- and carboxyl termini of the proteins (Figure 2), often representing polypeptide chains of 100 amino acids and more [7,10,11,18-22].

So far the largest sequence information could be obtained for flagelliform silk from *Nephila clavipes *(Figure 2B). This flagelliform silk protein is translated from a ~15.5 kb mRNA transcript originating from a 30 kb *Flag *locus [9,23]. The coding sequence is divided into 13 exons. The NR amino-terminal region is split between exons 1 and 2. All of the other exons are found to encode exactly one repeating unit, built from the described motifs (Figure 2B). The final exon 13 in addition includes the NR carboxyl-terminal region.

On the basis of several studies, the major categories of peptide motifs in spider silk proteins have been assigned structural roles [24-28]. The GPGXX motif has been suggested to be involved in a β-turn spiral, probably providing elasticity, based on structures of comparable proteins [29-32]. If elasticity is due to GPGXX β-spirals, then this motif should be found in the more elastic silks. Flagelliform silks, which show the highest elasticity with more than 200 %, consist of contiguous repeats of this motif for at least 43 times in each repeating unit (Figure 2B). The only non-flagelliform silk proteins with GPGXX motifs are MA proteins MaSp2, ADF-3, and ADF-4, which also display some viscoelasticity. In accordance to the lower elasticity of dragline silk in comparison to flagelliform silk the number of tandemly arrayed repeats depicts at most 9 concatenated GPGXX motifs before interruption by another motif [1,21]. Alanine-rich motifs contain typically 6–9 alanine residues and have been found to form crystalline β-sheet stacks leading to tensile strength [6,24,25,12]. The MA and MI silks are both very strong, and at least one protein in each silk (there are always pairs) contains the A_n _or (GA)_n _motif. Interestingly, this motif is not found in flagelliform silks. A glycine-rich 3_1_-helix is adopted by the GGX motif forming an amorphous matrix that connects crystalline regions and that provides elasticity [26,33,34]. The postulated GGX motif is widely distributed and this motif can be found in MA, MI and flagelliform silks (Figure 2A). Several groups have suggested that the motifs GPGXX and GGX might be involved in forming an amorphous matrix, which would provide the elasticity of the fiber. The spacers contain charged groups and separate the iterated peptide motifs into clusters. Non-repetitive termini are common to all sequenced MA, MI and flagelliform silks belonging to the Araneoidea family with highly conserved carboxyl-terminal sequences [19,35,36]. The structural impact of the spacer and terminal regions is so far undetermined [37]. Recent findings on single NR-regions of ADF-3 and ADF-4 (without additional repeating units) revealed a secondary structure comprising α-helices as determined by Circular Dichroism and they seem to retain this structural feature in proteins that additionally contain repeating units [36]. It can be speculated that the α-helical NR carboxyl-termini might play a crucial role during assembly of the silk fiber [19,36,38].

### Natural spider silk assembly

Silk assembly *in vivo *is a remarkable process. For instance, dragline silk proteins are stored at concentrations up to 50 % (w/v) in the respective glands [39]. This highly concentrated protein solution forms the silk dope (spinning solution), which displays properties of a liquid crystal [40-42]. Therein, the polyalanine motifs are thought to adopt an α-helical conformation, while the glycine-rich motifs form either β-turns or random coil conformation [39,43,44].

Thread assembly is initiated during a passage of the silk dope through the spinning duct accompanied by extraction of water, sodium and chloride [45,46]. Simultaneous secretion of potassium and hydrogen ions into the lumen of the duct lowering the pH from 6.9 to 6.3 is thought to initiate partly unfolding of the proteins by disrupting their water shell and altering coulombic forces [42,45-48]. The silk proteins are thought to extend somewhat, align and get packed much closer in the extensional flow-field of the draw-down taper found in the distal part of the duct. As the hydrophobic polyalanine segments of the silk proteins align and are drawn closer together by extensional flow, they are exposed to an increasingly hydrophobic environment, which might trigger their conversion from an α-helical to a β-pleated structure resulting in the formation of numerous interchain hydrogen bonds. The latter would act as multifunctional crosslinks at nodes between the more mobile glycine-rich segments. Thus the assembly of the thread can be seen as a liquid-crystalline phase transition involving separation into polymer-rich and solvent-rich phases [47].

While some aspects of spider silk assembly have been unraveled, the contribution of the individual silk proteins to the assembly process still needs to be resolved in more detail. Comparative studies of the two major dragline silk proteins of *Araneus diadematus*, ADF-3 and ADF-4, revealed that, although their amino acid sequences are rather similar [5], they display remarkably different solubility and assembly characteristics: While ADF-3 is soluble even at high concentrations [49], ADF-4 is virtually insoluble and self-assembles into filamentous structures under specific conditions [50]. At a closer look, the different solubilities of ADF-3 and ADF-4 could be explained by the hydrophobicities of the two proteins. The hydrophilic ADF-3 interacts favourably with the aqueous solvent and thus remains soluble under most conditions. In contrast, the more hydrophobic ADF-4 favours interactions with other protein molecules and thus tends to aggregate. Interestingly, all pairs of dragline silk proteins from different spider species display a common distinct distribution of hydrophobicity. In direct comparison, MaSp1 / ADF-4 proteins generally display relatively high hydrophobicity, while the corresponding MaSp2 / ADF-3 partner protein is more hydrophilic [50].

### Applications for spider silks

Laboratory-scale production of spider silk would initiate a new generation of ecological materials. Spider silk is for instance a promising tool with broad usability in medical devices. In the middle ages spider webs were used as wound dressing – some reports are even dated back to ancient Greek and Roman cultures. Silkworm silk does not cause allergic reactions and it is thought that spider silk behaves similarly [51]. The unmatched toughness of spider silk would allow to improve several medical products such as wound closure systems, band-aids, and extremely thin sutures for neurosurgery. Additionally, spider silks can be further used in artificial ligaments and tendons for durable implants. High performance fibers built from spider silks can be employed in several technical and industrial applications. In addition to specialty ropes and fishing nets, spider silk can be utilized for parachutes, ballistic applications (body armor), sporting goods, textiles, and lightweight constructions for airplanes [52,53]. Therefore, one day industrially produced spider silk could out-compete man-made fibers.

### Production of recombinant spider silk proteins

Recombinant production of spider silk proteins has been complicated by the highly repetitive nature of the underlying genes, by their high *gc*-content, by the length of the constructs, and by the specific codon usage of spiders. In first studies, *in vitro *translation of mRNA from excised major ampullate glands of *Nephila clavipes *was performed using tRNA from *E. coli*, but translation was discontinuous [14,54]. In the era of recombinant proteins and genetic engineering one would envisage to easily produce spider silk proteins (mainly from draglines) in microbes or cell culture. Unfortunately, no dragline silk gene has been cloned in its entirety and only sequence data from the 3' end of partial cDNA clones of dragline genes from *Nephila clavipes *and *Araneus diadematus *and other spiders have been reported [10,11,20-22]. Therefore, all recent studies used partial cDNA constructs of dragline silk genes to produce recombinant silk proteins in *E. coli *[55], in MAC-T (bovine) and BHK (hamster) cells [49], or in insect cell lines from *Spodoptera frugiperda *using the baculovirus expression system [50]. The most promising expression system seems to be the baculovirus system, since it was possible to efficiently produce dragline silk components at a high yield.

### Designing of synthetic spider silk proteins

Cloning strategies for designing genes for bacterial, yeast or plant expression have been developed to produce recombinant silk-like proteins closely resembling natural dragline [29,36,56-62] or flagelliform silk proteins [63]. Since gene manipulation and amplification of spider silks is difficult by PCR due to the repetitive nature of silk genes, cloning strategies involved engineering of synthetic DNA modules. These modules were optimized for the codon usage adapted by the corresponding expression host. The use of synthetic modules constructed from small size oligonucleotides repeats has allowed control over primary gene and protein sequence and final protein size. Tobacco, potatoes, the yeast *Pichia pastoris *and mainly *E. coli *have been utilized as expression hosts for synthetic genes yielding proteins with up to 150 kDa [29,56-62]. Unfortunately, expression levels from the synthetic genes have been low and mostly the recombinant silk proteins represented only up to 5% of the total protein in the cell [13]. Although once production levels of up to 1000 mg/l of cell culture have been reported [57], large losses in yield are encountered during purification due to precipitation and non-specific interactions. For the microbial expression systems, yields of purified proteins have been generally in the 10 to 40 mg/l range (>90% purity) [[55,56,59,60], summarized in [13]].

**Figure 3:**
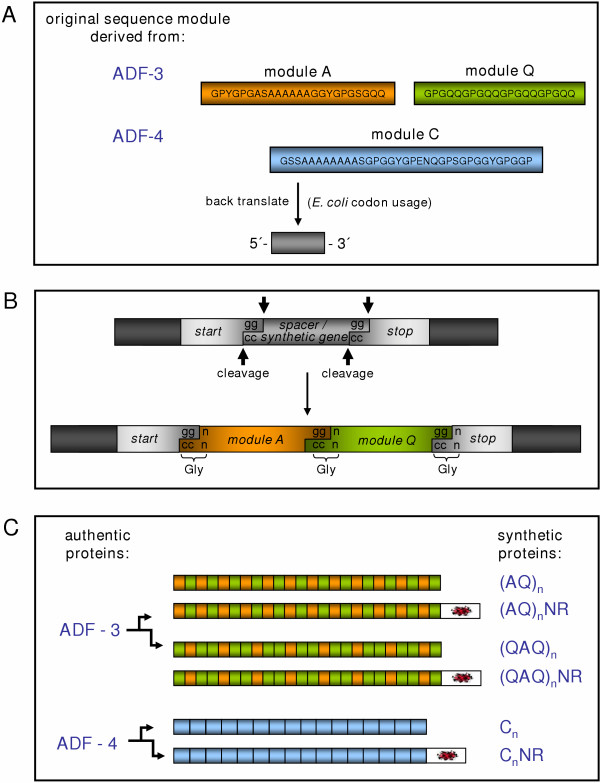
**Cloning strategy for constructing synthetic spider silk genes. **(A) Amino acid sequences of designed silk modules were derived from dragline silk proteins ADF-3 and ADF-4 and back translated into nucleotide sequences using the bacterial codon usage. (B) The cloning cassette comprised restriction sites required for module insertion and multimerization. During gene construction a spacer region was replaced by modules and module multimers. The first codon of each module (*ggn*) determined the "linking" amino acid glycine. (C) Single modules were connected resulting in controlled assembly of synthetic genes. To study different length repeat units, one or two Q modules were combined with one A module to obtain (AQ) or (QAQ). These repeat units were multimerized to generate synthetic genes coding for the repetitive proteins (rep-proteins) (AQ)_n _and (QAQ)_n_. The repetitive part of ADF-4 is generally composed of a single consensus module termed C, which was multimerized to obtain the rep-protein C_n_. Additionally, carboxyl-terminal non-repetitive (NR)-regions from the natural genes were amplified by PCR and optionally linked with the synthetic genes [50].

In a recent study, genes coding for spider silk-like proteins were generated using a cloning strategy, which was based on a combination of synthetic DNA modules and PCR-amplified authentic gene sequences (Figure 3) [36]. This approach was in contrast to previous protein designs, which did not include the carboxyl-terminal NR-regions that are found in all dragline silks. The dragline silk proteins ADF-3 and ADF-4 [10] from the garden spider *Araneus diadematus *were chosen as templates for the synthetic constructs. A seamless cloning strategy [64] allowed controlled combination of different synthetic DNA modules as well as authentic gene fragments. A cloning vector was designed comprising a cloning cassette with a spacer acting as placeholder for synthetic genes [36] (Figure 3B).

To mimic the repetitive sequence of ADF-3, two modules have been designed. The sequence of one module, termed A, was derived from the poly-alanine containing consensus sequence of ADF-3 (Figure 3A). The sequence of a second module termed Q contained four repeats of the GPGQQ motif. In a first cloning step the spacer region of the cloning vector was replaced by one of the synthesized DNA modules. Subsequently two modules could be joined in a site-directed way. To study different length repeat units, one or two Q modules were combined with one A module to obtain (AQ) (Figure 3B) or (QAQ) (Figure 3C). The complementary 3'-single strand extensions *gg *(sense) and *cc *(antisense) were used for connecting two modules (Figure 3B). Thus the DNA sequence required to link two modules was confined to a glycine codon (*ggn*). Glycine is naturally abundant in spider silk proteins (~30%), therefore modules could be designed to match authentic amino acid sequences. Since the arrangement of the cloning cassette's elements remained unchanged upon cloning, repeat units could be multimerized to generate synthetic genes coding for the repetitive proteins (rep-proteins) (AQ)_12 _and (QAQ)_8 _(Figure 3C).

The repetitive part of ADF-4 is generally composed of a single conserved repeat unit displaying only slight variations. These variations were combined and one consensus module termed C has been designed (Figure 3A), which was multimerized to obtain the rep-protein C_16 _(Figure 3C).

ADF-3 and ADF-4 both display NR-regions at their carboxyl termini, comprising 124 and 109 amino acids respectively. Gene sequences coding for these regions were amplified by PCR, and codons problematic for bacterial expression were changed to more suitable codons by site directed mutagenesis. In the described system, all synthetic genes could be combined with the appropriate authentic NR-regions. Additionally NR3 and NR4 could be expressed individually. All constructs could be purified by a heat step followed by an ammonium sulfate precipitation [36], which has been employed in previous studied for purifying spider silk proteins [35,62].

Based on this protein engineering approach, which combines synthetic repetitive sequences with authentic NR-regions, proteins closely resembling authentic silk proteins could be produced at high yields. Bacterial production in Erlenmeyer flasks yielded similar protein amounts for all constructs. Yields of individual preparations ranged from 10 to 30 mg of purified protein per liter of culture medium. Fermentation of cells increased the yield of purified protein to 140 and 360 mg/l (purity >95%). Therefore, the established bacterial expression system provides the basis for cost-efficient large scale production of spider silk-like proteins.

### Assembly of recombinant spider silks

**Figure 4:**
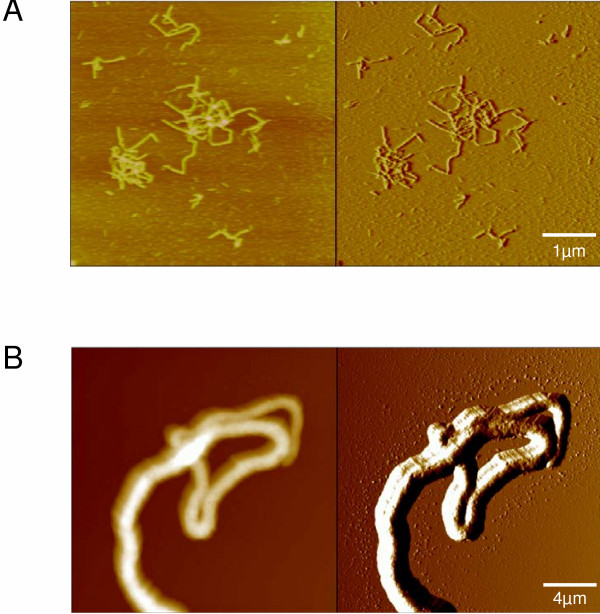
**Morphology of self-assembled fibers of recombinant spider silk proteins. **Images of nanofibers of the synthetic protein C_16 _assembled *in vitro *(A) and of ADF-4 fibers assembled in insect cells (B) were obtained by atomic force microscopy (AFM). The left images depict the height information, the right images the deflection. The average height of the C_16_-nanofibers is 2 – 4 nm, the height of the visualized ADF-4 fiber is 0.7 μm.

One important feature of spider silk proteins is their storage at high protein concentrations (up to 50% w/v) in the dope without apparent aggregation or assembly. However, spider silk proteins can rapidly assemble into highly stable fibers when needed. The determination of solubility and self-assembly of recombinant spider silk proteins is therefore important to create commercially available silk fibers. For instance, pH-shifts are involved in natural silk assembly, but the exact function of acidification during spider silk assembly has not yet been determined. It seems likely that negatively charged groups are protonated reducing the net charge of spider silk proteins. Phosphoryl groups of phosphorylated amino acid residues, which have been detected in dragline silk [65], display pK_A_-values [66] near the range of the pH-shift observed during the spinning process and thus could be involved in triggering silk assembly. Therefore, changes in pH can be used to initiate assembly of recombinant spider silk proteins [36]. Interestingly the intracellular pH 6.3 of *Sf9 *cells (derived from the fall armyworm *Spodoptera frugiperda*) used for producing ADF-4 corresponds to the pH in the spinning dope prior to silk thread assembly [50]. These pH conditions, among additional factors, might be involved in initiating aggregation of ADF-4 within the cytosol of *Sf9 *cells [50]. Surprisingly, investigating the aggregates in *adf-4 *expressing cells revealed filaments that coiled throughout the cytoplasm, whereby most of the cells contained only one or few filaments. In contrast, *Sf9 *cells infected with control viruses or the *adf-3 *encoding virus never produced such filaments. The diameter of the ADF-4 filaments (0.2 – 1.5 μm) was in the range of native dragline silks (1.0 – 4.0 μm), but length of the filaments formed in the *Sf9 *cells seemed to be constrained by the volume of the cells, making them too short for mechanical force measurements. Strikingly, the purified ADF-4 filaments (Figure 4B) showed a similar morphology and chemical stability in comparison to natural dragline silk threads of *Araneus diadematus *[50].

Phosphate, like other lyotropic ions, is known to increase the surface tension of water, promoting hydrophobic interactions [67]. In the case of spider silk proteins it is likely that the addition of phosphate initiates interactions between the hydrophobic poly-alanine motifs, causing the aggregation of the proteins. Accordingly aggregation of polyalanine-rich proteins is pronounced in comparison to synthetic silks which contained one third less poly-alanine motifs [36]. Strikingly, recombinant spider silk proteins are highly soluble in most aqueous solutions, but form nanometer-sized fibers upon addition of methanol, phosphate or other suitable ions (Figure 4A).

### Artificial spinning of spider silks

A remaining critical step concerning commercial production of silk fibers is the successful spinning of recombinant proteins into fibers resembling the natural silks in their microstructure and in their mechanical properties, which are outstanding by any measure. Besides the chemical parameters discussed above, several mechanical parameters play important roles in generating silk. To draw silk under natural spinning conditions, spiders attach their dragline to an object with glue from the piriform glands, before drawing the silk out by moving away or by descending and using their weight to draw the silk. It is common practice to take advantage of the drawing process by the forced silking of captive animals to collect silk for experiments. Analysis of the differences between naturally and forcibly spun dragline silk provided evidence for discrepancies in their material properties [44,68,69]. Forced spinning under spinning speeds ranging from 0.1 to 400 mm/s and temperatures ranging from 5 to 40°C revealed dramatic differences in strain at breaking, breaking energy, initial Young's modulus and point of yielding [70]. Therefore, in case of spinning recombinant silk proteins *in vitro *several aspects have to be taken into account to gain materials with expected properties.

Several attempts are reported in the literature and even more have been performed to wet-spin recombinant spider silk proteins. In a first attempt, microfabricated spinnerets were constructed using silicon microfabrication methods [71-73]. These spinnerets allowed for the production of meters of silk fibers from solutions containing as little as 10 mg of protein. First the spinneret was validated and tested by producing fibers from dissolved silk from the silkworm *Bombyx mori *[71], before solubilized dragline silk from *Nephila clavipes *was wet-spun [72]. The diameters and mechanical properties of the regenerated silkworm silks converged the native silk ones. However, the wet-spun spider silks exhibited diameters of about 40 μm compared to the natural fiber diameter of 2.5 to 4.0 μm with mechanical properties that did not match the natural ones [72]. Other attempts of wet-spinning revealed fiber diameters of approximately 10 – 60 μm [49,74]. These fibers were subjected to either single or double postspinning draw, first in 70 to 80 % methanol (single and double draw) and then in water (double draw only) to increase their mechanical properties. Fibers subjected to higher draw ratios displayed greater toughness, tenacity, and modulus values [49]. However, even the best values obtained by such technique were in the range of the regenerated *Nephila *fibers [72], but lower than the reported values for natural dragline silks [2].

## Perspectives

### Engineering of precision fibers

**Figure 5:**
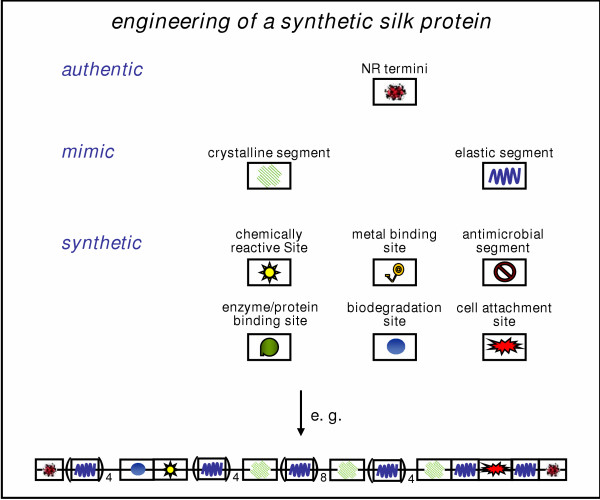
**Engineering of a synthetic silk protein. **The future objective of silk engineering might not be to prepare identical copies of natural silks, but rather to capture its key structural and functional features in designs. The soluble synthetic silk should be able to assemble into protein fibers with desired properties, which includes the possibility to specifically functionalize the fiber surface e.g. by chemical cross-linking with biologically or chemically active groups. Synthetic silk engineering could be accomplished by assembling modules either originating from authentic genes, mimicking silk motifs or being entirely synthetic with defined functionality. The follow-up strategy would be to modify the resulting monomeric soluble protein either with the desired functionality prior to assembly into fibers, or to incorporate a reactive group that will subsequently permit the conjunction with desired functionality after the fibers have assembled.

The future objective might not be to prepare identical copies of natural silk fibers, but rather to capture its key structural and functional features in designs that could be useful for engineering applications (Figure 5). Using "protein engineering" based on knowledge achieved from investigations of the natural silks, artificial proteins can be designed that allow bacterial synthesis at high yields [75]. The soluble synthetic silk would be able to assemble into protein fibers with desired properties, which includes the possibility to specifically functionalize the fiber surface by chemical cross-linking with biologically or chemically active groups. Such protein fibers could be optimized by additional protein engineering in order to gain fibers that allow the formation of interconnected nano- or micrometer-scale networks, which are capable of various biological, chemical or physical processes (e.g. enzymatic reactions, chemical catalysis, electronic signal propagation, etc.) (Figure 5). The main strategy would be to modify the monomeric proteins either with the desired functionality prior to assembly into fibers, or to incorporate a reactive group that will subsequently permit the conjunction with desired functionality after the fibers have assembled. Protein fibers could for instance be covalently linked with external units through chemically specific amino acid side chains (e.g. SH-groups of cysteines) [76-78].

Since the physical and chemical properties of bio-polymers and their assembly processes depend on the amino acid composition of the underlying polypeptide, engineering "synthetic" proteins with specific structural features will create a new class of fibrous proteins. However, to design new biomaterials based on spider silk, all properties of the underlying proteins have to be analyzed and in the best case successfully mimicked [35]. Therefore, the crucial design features of both the feedstock of the dope and the spinning process have to be closely adopted, which would allow for managing the commercial production of new materials.
